# Supplementary figures and images for: Beyond its preferential niche: Brucella abortus RNA down-modulates the IFN-γ-induced MHC-I expression in epithelial and endothelial cells
Source: PLoS One. 2024 Jul 9;19(7):e0306429. doi: 10.1371/journal.pone.0306429 (PMC11232970; doi:10.1371/journal.pone.0306429)

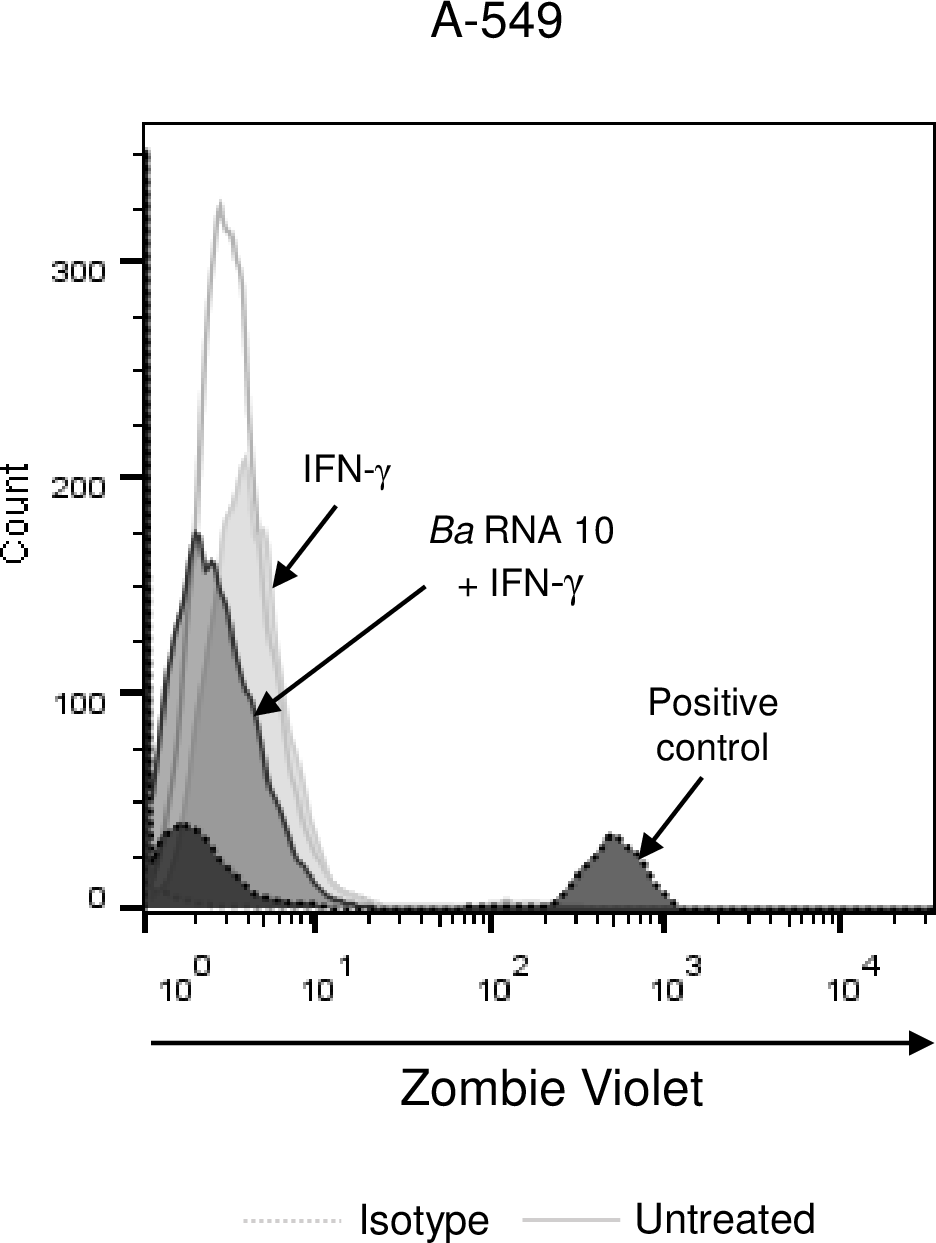

Supplement: S1 Fig — A-549 were attached on a 48-well plate for 24 h. Then, the cells were stimulated with Ba RNA (10 μg/ml) for 48 h in the presence of IFN-γ. They were then stained with Zombie Violet™ solution to check the viability of the treated cells. A set of heat-killed cells were used as a positive control. This is a representative flow cytometry histogram of the results show in Fig 2. (TIF) [file pone.0306429.s001.tif]

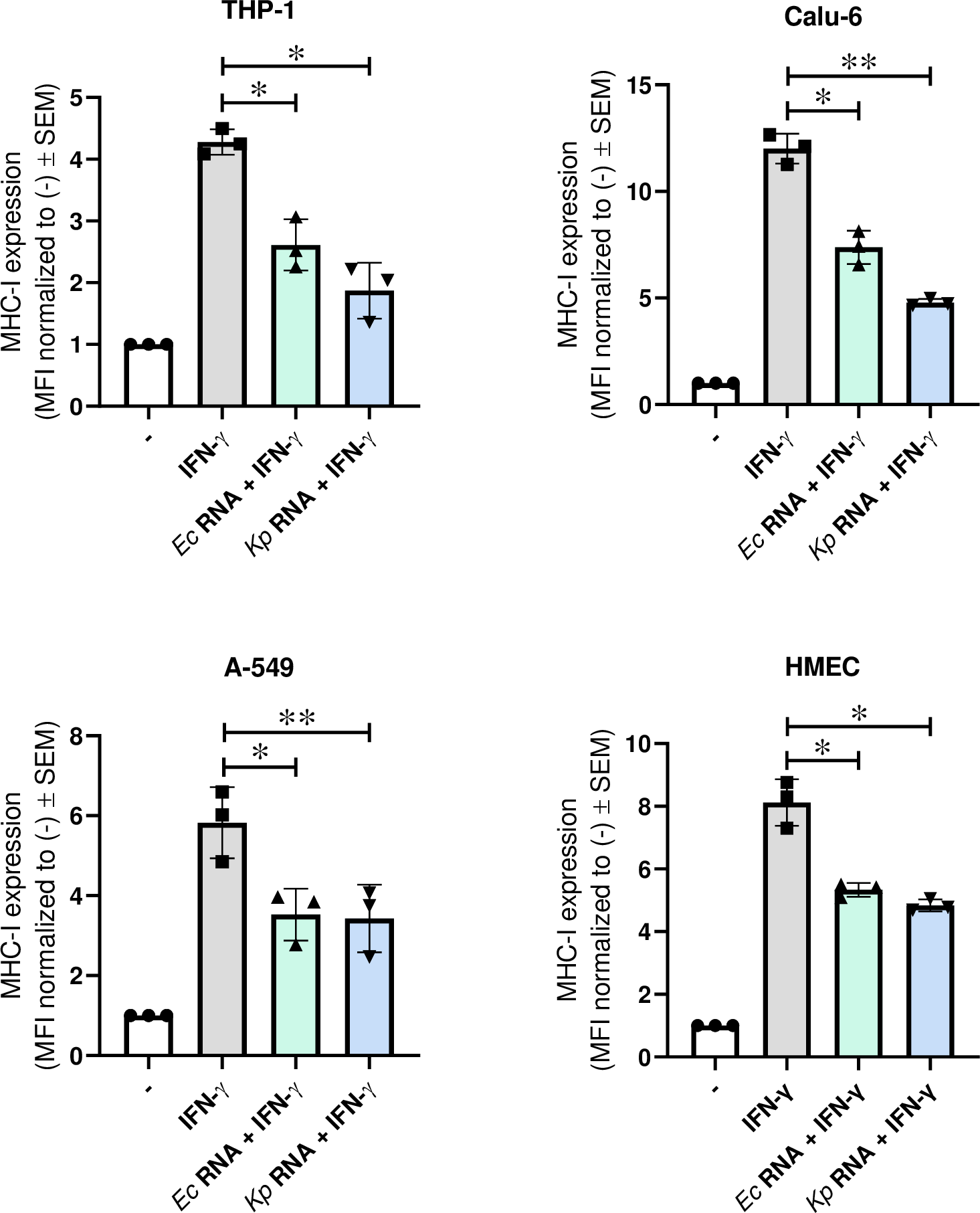

Supplement: S2 Fig — THP-1, Calu-6, A-549 and HMEC were attached on a 48-well plate for 24 h. Then, cells were stimulated with Ec or Kp RNA (10 μg/ml) in the presence of IFN-γ. After 48 h, flow cytometry was used to detect MHC-I expression. IFN-γ-treated cells were used as a positive control. Bars represent geometric means normalized to untreated cells ± SEM from three independent experiments. MFI, mean fluorescence intensity. -, untreated cells. *P<0.05; **P<0.01 vs IFN-γ-treated cells. (TIF) [file pone.0306429.s002.tif]

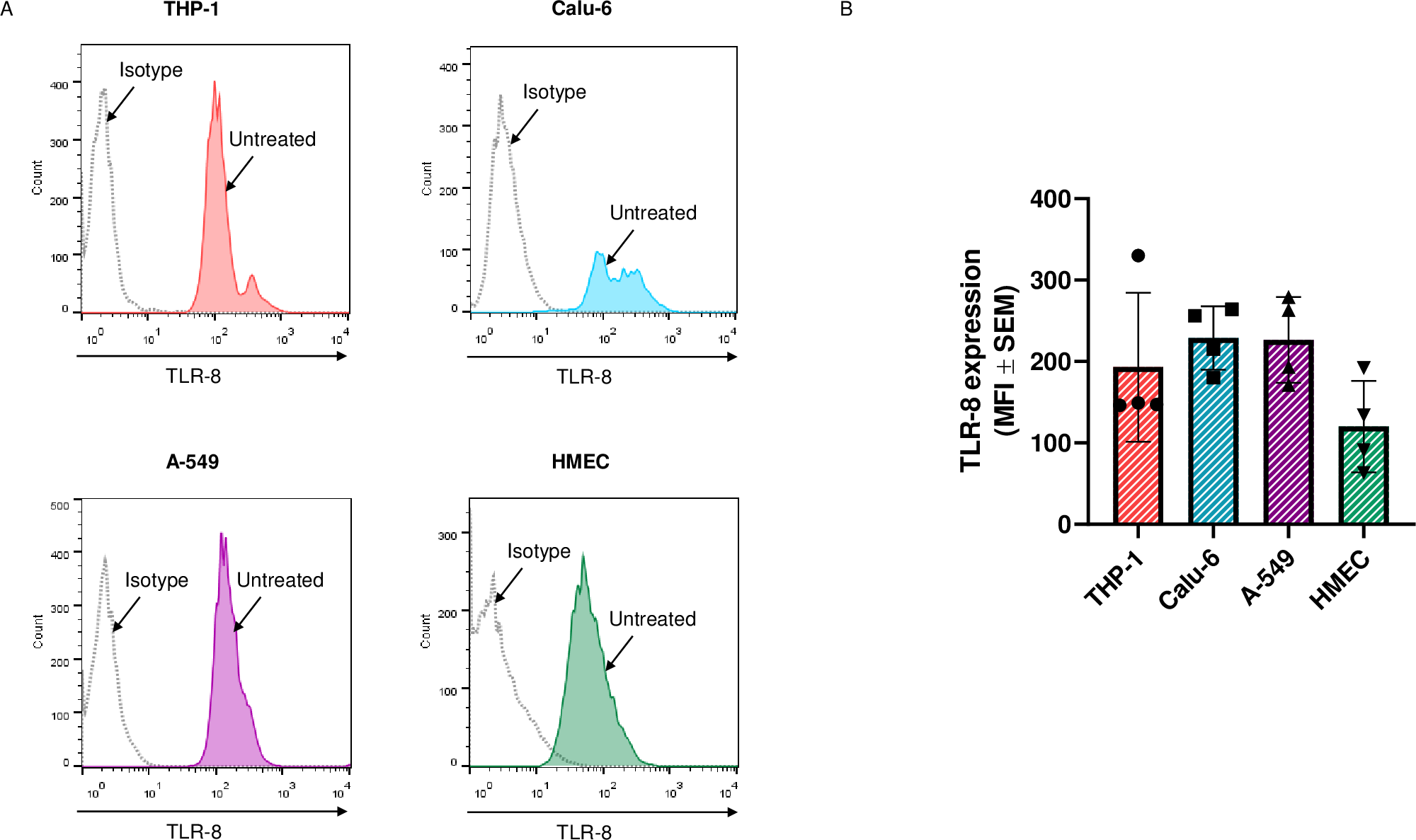

Supplement: S3 Fig — THP-1, Calu-6, A-549 and HMEC cells were attached on a 48-well plate for 24 h. Then TLR-8 expression was determined by flow cytometry. (A) These are representative flow cytometry histograms of results shown in panel B. (B) Bars indicate the geometric means ± SEM of four independent experiments. MFI, mean fluorescence intensity. (TIF) [file pone.0306429.s003.tif]
